# The effects of protein isolates and hydrocolloids complexes on dough rheology, physicochemical properties and qualities of gluten‐free crackers

**DOI:** 10.1002/fsn3.266

**Published:** 2015-09-01

**Authors:** Natthakarn Nammakuna, Sheryl A. Barringer, Puntarika Ratanatriwong

**Affiliations:** ^1^ Department of Agro‐IndustryFaculty of Agriculture Natural Resources and EnvironmentNaresuan UniversityPhitsanulok65000Thailand; ^2^ Department of Food Science and TechnologyThe Ohio State University110 Parker Hall2015 Fyffe RoadColumbusOhio43210

**Keywords:** Gluten‐free rice crackers, hydroxypropylmethylcellulose, pea protein isolate, soy protein isolate, whey protein isolate, xanthan gum

## Abstract

To understand the suitability of protein‐hydrocolloid complexes as replacement for wheat protein in rice crackers, and the effect of protein source, carboxylmethylcellulose (CMC) and hydroxylpropylmethylcellulose (HPMC) at 1.0%, 1.5%, and 2.0% w/w, and 0.25%, 0.50%, and 0.75% w/w of xanthan gum (XN) were added to flour‐blendedrice crackers (FF). A variety of protein isolates was added to 2.5%, 5.0%, and 10% w/w combinations of protein isolates and hydrocolloids were investigated. The controls were FF, 100% rice crackers (RF), and wheat crackers (WF). About 1.5% CMC samples had the closest hardness to WF, followed by 0.5%XN and 1.5%HPMC, and 0.5%XN crackers had the highest moisture content and water activities followed by 0.75%XN, 1.5%CMC, and 1.5%HPMC. Increasing % of hydrocolloids also increased puffiness. Protein isolate crackers had higher moisture content and water activity. Protein isolates improved puffiness. Whey protein improved elasticity, while hydrocolloids added to leguminous protein increased loss tangent.

## Introduction

Celiac disease affects about 0.2–1.0% of the world population and the number is steadily increasing worldwide (Abdel‐Aal 2009; Toft‐Hansen et al. 2014). Patients who have celiac disease are unable to consume products made from wheat flour (WF). The replacement of gluten in a cereal‐based food system poses a major technological challenge due to gluten's structure‐forming capacity. Gluten substitutes must be able to form cohesive elastic dough that can be baked into a food product with pleasant taste and acceptable texture (Abdel‐Aal 2009). The removal of wheat proteins from gluten‐free cracker products causes significant changes in the volume, brittleness, and rheological properties.

Rice flour (RF) has been used as the basic ingredient in gluten‐free bread because it lacks gluten and contains low levels of sodium and high amounts of easily digested carbohydrates (Gallagher et al. 2002). However, rice proteins have relatively poor functional properties for food processing. Due to their hydrophobic nature, rice proteins are insoluble and unable to form the viscoelastic dough necessary to hold the carbon dioxide produced during proofing of yeast‐leavened bread‐like products (He and Hoseney 1991). Flour blends (FFs) consisting of pregelatinized starch have been traditionally used as a replacement for WF in gluten‐free crackers, however, the crackers produced with these blends have been deemed to be of lower eating and overall organoleptic quality than wheat crackers. Proteins are added to gluten‐free applications to increase elastic modulus by cross linking, to improve perceived quality, to improve structure with gelation, and to aid in foaming (Crockett et al. 2011). The high quality of dairy ingredients‐containing breads was attributed to the ability of the dairy ingredient to form a network similar to gluten. Hydrocolloids are used in a wide range of food applications to impart texture and appearance as well as to improve product stability. In the baking industry, hydrocolloids are of increasing importance as bread‐making improvers, and help increase dough properties such as water absorption, gas retention, and improve product properties such as texture and to retard starch retrogradation (Bárcenas and Rosell 2005; Lazaridou et al. 2007). Hydroxyl propylmethyl cellulose (HPMC) were reported to provide dough stability during proofing with increasing gas retention by adding strength to gas cells, resulting in better gas retention and higher product volume (Bell 1990). In bread crumbs, addition of xanthan help increase the hardness of the bread crumb by acting as a thickener (Rosell et al. 2001). Carboxylmethylcellulose (CMC) and HPMC help increase the crispness of partial wheat‐substitute crackers by reducing the hardness and increase the fracture of crackers (Nammakuna et al. 2009).

Despite much research into gluten‐free baked products, information on gluten‐free crackers made from RF is rarely found. No research on optimization of hydrocolloids or their combination on the qualities of gluten‐free crackers from RF has been reported. Available information is still limited, in particular research on suitable types of protein isolates and the optimum amounts to add. An understanding of the effects of combinations of protein isolates and hydrocolloids on cracker properties is still needed. Thus, this research aims to understand the relationship between the type, concentration, and interaction of hydrocolloids and protein isolates on the overall quality and rheology of gluten‐free cracker products made from rice FF.

## Material and Method

### Gluten‐free cracker preparation from rice FF

The gluten‐free cracker formulation was created by using the mixed FF with RF as major raw material to completely substitute WF in the cracker formulation. The mixed FF consisted of three flour types including low‐amylose content RF (Chaijalearn Co., Ltd., Bangkok, Thailand), waxy RF (Jewhoksang Co., Ltd., Lampang, Thailand) and tapioca pregelatinized starch (Bangkok Starch Co., Ltd., Bangkok, Thailand). This mixed‐FF without addition of either hydrocolloids or protein isolates was used as control for treatment samples whereas 100%RF and 100%WF were also used as negative control and benchmark, respectively.

The basic ingredients for making crackers for all formulations were salt, sugar, milk powder, palm oil, glucose syrup, baking powder, margarine, dry yeast, ammonia powder, lecithin, corn flour, and tapioca flour. These ingredients were used as basic ingredients. Firstly, flour, sugar, yeast, and water were blended in a mixer (Kitchen‐Aid model 5SS, St. Joseph, MI, USA) for 5 min, and then the rest of the ingredients were added. The mixture was blended for 7 min before proofing the dough at 25–30°C for 60 min. After proofing, the dough was kneaded, sheeted, layered, cut into a cracker size of 2.5 × 5 cm, and subsequently baked in the oven at 180–200°C for 15 min. Baked crackers were then cooled, packed in sealed polyproplylene bags, and stored at room temperature before further experiment.

### Experimental design

Three types of hydrocolloids (Bronson & Jacobs International Co., Ltd., Bangkok, Thailand) including CMC and HPMC and xanthan gum (XN) were added to FF. These hydrocolloids were added at different concentrations. HPMC and CMC were added at 1.0%, 1.5% and 2.0%, respectively; XN was added at 0.25%, 0.50%, and 0.75% (total RF basis) to mixed FF. Three types of protein isolates including, soy protein isolate (F.A. Unity Co., Ltd, Bangkok, Thailand), pea protein isolate (Roquette Singapore PTE, LTD, Bangkok, Thailand), and whey protein isolate (Grande Custom Ingredients Group, Lomira, WI 53048, United States) were added at 2.5%, 5.0%, and 10.0% (total RF basis) on mixed FF. The qualities of all treatment samples were determined according to 2.3 and compared to controls. Moreover, the impact of combinations of protein isolates and hydrocolloids, added at optimum level to FF, on gluten‐free rice crackers were also investigated. The qualities of all treatment samples were determined according to Section Quality determination of rice crackers and compared with the three controls.

### Quality determination of rice crackers

#### Moisture content and water activity (*a*_w_) determination

The moisture content and the water activity (*a*_w_) of samples were determined in three replicates using a moisture meter model Sartorius MA40 (Sartorius, Inc., Goettingen, Germany) and a water activity meter model Novasina RS 200 (Novasina, Axair Ltd., Pfaffikon, Switzerland), respectively.

#### Texture properties

Texture was determined in ten replicates using a Texture Analyzer model QTS25 (Brookfield Engineering Labs., Inc. Middleboro, MA 02346 U.S.A.) equipped 25 kg load cell. The three‐point bend fixture test method was used. Texture profile analysis was performed. The textural characteristics were expressed in terms of hardness (the height of the force peak on the first compression cycle [first bite]), cohesiveness, chewiness, and springiness (the spring back after it has been deformed during first compression) of gluten‐free rice crackers. Each sample was analyzed in 10 replicates.

#### Color measurement

Cracker samples were measured for color in CIE system (L*, a*, b*, hue angle, and chroma) by a color reader model CR‐10 (Konica Minolta sensing Inc., Osaka, Japan). The analysis was performed four replicates for each sample.

#### Rheological properties of gluten‐free rice cracker

The dough rheological measurement was studied by using the dynamic oscillatory test. The test was performed by a controlled stress–strain rheometer (Physica MCR 301, Physica/Anton Paar, Germany), using a parallel‐plate geometry (PP25/TG 6866) with plate diameter and plate gap of 25 and 2 mm, respectively. A frequency sweep test provided the information of dough rheological changes including the structure, molecular structure, and viscoelastic behavior (Angioloni and Rosa, 2007). The measurements were conducted at 25°C by using a frequency sweep from 0.1 to 10 Hz at 0.1% strain.

#### Puffiness (%)

Thicknesses of the cracker before and after baking were measured by vernier calipers in five replicates then the sample puffiness (%) was calculated from the difference of cracker thicknesses as shown in Eq. (1).

(1) $$\%\;\text{puffiness}=\left(\frac{\text{thickness of baked cracker}-\text{thickness of cracker dough}}{\text{thickness of cracker dough}}\right)*100.$$

#### Scanning electron microscope

The dough microstructure was studied using a scanning electron microscope (SEM) Model 1455VP (Leo Electric Systems, Cambridge, UK). Prior to the SEM study, cracker dough samples were cut to size 10 × 10 mm, freeze dried, and kept in a desiccator until further use. Dough samples were mounted on a slide and separately placed on a sample holder using double‐sided scotch tape. The internal structure was upward facing and sputter‐coated with gold (2 min, 2 mbar) before being transferred on to a microscope where it was observed in vacuum at an accelerating voltage of 5 kV.

### Statistical analysis

The qualities of samples were analyzed in two replicates. The experimental design used in this research was a Completely Randomized Design. All treatments were done in duplicate. Data were statistically analyzed by Ducan's Multiple Range test at 95% confidence level.

## Results and Discussion

### Effect of hydrocolloids on physicochemical properties of gluten‐free dough and crackers

All the treatment samples had significant differences in moisture content, water activity (*a*_w_), and puffiness (*P *≤* *0.05) as observed in Table 1.

**Table 1:** Effect of hydrocolloids on physicochemical properties of gluten‐free rice cracker

Samples	Moisture content (%)	Water activity (*a*_w_)	Puffiness (%)
WF	5.99 ± 0.16^c^	0.343 ± 0.005^c^	84.91 ± 2.58^a^
RF	5.13 ± 0.32^g,h^	0.302 ± 0.001^e^	20.08 ± 1.84^i^
FF	5.41 ± 0.16^e,f^	0.326 ± 0.007^d^	37.98 ± 4.29^g^
0.25%XN	5.00 ± 0.17^h^	0.276 ± 0.015^f^	42.60 ± 1.62^f^
0.5%XN	7.16 ± 0.21^a^	0.430 ± 0.000^a^	46.06 ± 2.20^e,f^
0.75%XN	6.21 ± 0.26^b^	0.365 ± 0.003^b^	43.99 ± 1.64^f^
1.0%CMC	5.08 ± 0.18^g,h^	0.277 ± 0.006^f^	59.28 ± 1.24^c^
1.5%CMC	6.08 ± 0.17^b,c^	0.363 ± 0.001^b^	73.03 ± 2.11^b^
2.0%CMC	5.34 ± 0.09^f^	0.295 ± 0.002^e^	52.49 ± 0.98^d^
1%HPMC	5.23 ± 0.28^f,g^	0.342 ± 0.001^c^	34.03 ± 2.59^h^
1.5%HPMC	5.70 ± 0.46^d^	0.344 ± 0.007^c^	44.17 ± 2.52^f^
2.0%HPMC	5.58 ± 0.17^d,e^	0.339 ± 0.001^c^	49.04 ± 2.59^e^

Different letters in each column indicate statistical differences (*P ≤ *0.05). Rice flour (RF), wheat flour (WF), and flour blend (FF) were controls and were made of 100%RF, 100%WF, and formulated FF, respectively.

Gluten‐free crackers were made with 0.5%XN and had the highest moisture content and water activity (*P *≤* *0.05). Gluten‐free crackers containing 1.5%CMC had moisture content closest to that of wheat crackers compared to other treatment samples. The addition of 1.0–2.0%HPMC lead to water activity values close to those of the wheat control (*P *≤* *0.05). Increasing the concentration of HPMC had no corresponding, statistically significant effect on the water activity value (*a*_w_) of the dough. The addition of 0.5%, 0.75%XN, and 1.5%, 2.0%HPMC increased both the water activity and moisture content of the finished baked product when compared to the hydrocolloids‐free control.

These results indicated the benefits of hydrocolloids as a dough improver as the hydrocolloids help increase the water‐holding capacity of samples due to the chemical structure of hydrocolloids and their interaction with the food ingredients (Rosell et al. 2001; Lazaridou et al. 2007).

**Figure 1:**
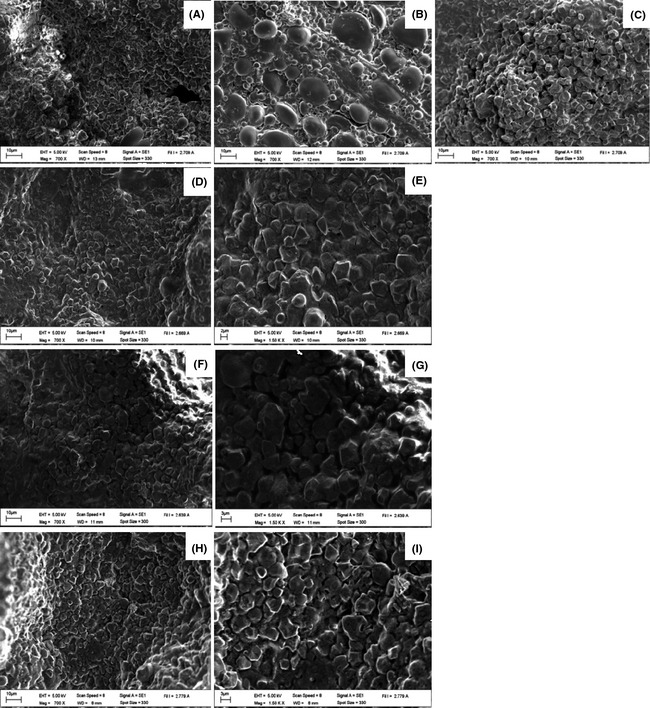
Effect of hydrocolloids on the dough microstructure of gluten‐free rice crackers. Rice flour (RF), wheat flour (WF), and flour blend (FF) were controls and were made of 100%RF (a), 100%WF (b), formulated FF (c), FF + 1.5%HPMC (×700) (d), (×1500) (e), FF + 0.5%XN (×700) (f), (×1500) (g), FF + 1.5%CMC (×700) (h) and (×1500) (i), respectively.

In addition, the barrier formed by hydrocolloids is illustrated in Figure 1. This barrier is formed by hydrocolloids near or at the surface during heating, which leads to reduction in water loss, thus increasing the final moisture content of the crackers. (Khalil 1999; Albert and Mittal 2002; Mellema 2003; Akdeniz et al. 2006).

The addition of hydrocolloids to the FF formula caused an increase in puffiness for all hydrocolloids tested with the exception of the 1.0% HPMC sample (Table 1). The treatment sample with CMC‐produced puffiness values was closest to those of wheat crackers. The extent of the puffiness increase was directly proportional to the usage level for some hydrocolloids such as HPMC, but was not significantly affected by the usage level for XN. Bell (1990) explained that HPMC gives some stability to the interface of a dough system during proofing and confers additional strength to the gas cells through baking, which increases gas retention and thus leads to higher volume. This was the same case as shown in Table 1, the dough of gluten‐free cracker with HPMC was stronger with better air retention thus an increase in puffiness of HPMC‐added crackers were observed. However, the optimum amount of HPMC was also crucial for puffiness because 1.0% HPMC did not have enough effect on gluten‐free crackers. In addition, Marco and Rosell (2008) found that the volume of rice bread increases with the addition of hydrocolloids except XN, and reported that high values of crumb porosity were obtained when 1.0%CMC and *β*‐glucans were added.

Samples treated with hydrocolloids had hardness and fracture force values lower than the WF control but higher than the RF and FF controls (Table 2). The increase in fracture force was directly proportional to the increase in usage level of the hydrocolloids for samples made with XN and CMC, Addition of hydrocolloids caused an increase in hardness values for all samples tested, so treated samples were less crumbly than RF which had the lowest hardness. The WF control had the highest fracture force which indicated that the texture of WF crackers were light crisp and brittle. This was caused by gluten known as the protein‐forming structure in wheat dough (Fig. 1).

**Table 2:** Effect of hydrocolloids on texture characteristics of gluten‐free rice cracker

Samples	Attributes			
	Hardness (g)	Cohesiveness	Chewiness (gmm)	Springiness (mm)
WF	325.25 ± 5.66^h^	0.64 ± 0.02	1.04 ± 0.01	0.06 ± 0.04
RF	156.20 ± 9.52^i^	0.12 ± 0.05	11.6 ± 1.86	0.51 ± 0.03
FF	396.67 ± 14.2^f^	0.02 ± 0.01	0.14 ± 0.02	0.01 ± 0.00
0.25%XN	481.69 ± 17.9^d^	0.21 ± 0.07	−102.01 ± 0.39	−2.81 ± 0.34
0.5%XN	367.73 ± 8.95^g^	0.35 ± 0.02	−126.02 ± 0.65	−2.11 ± 0.72
0.75%XN	653.18 ± 22.6^a^	6.61 ± 1.14	−124.19 ± 0.51	−2.45 ± 0.55
1.0%CMC	462.11 ± 9.99^d^	0.12 ± 3.09	−151 ± 5.22	−2.22 ± 0.06
1.5%CMC	386.90 ± 6.36^f,g^	1.15 ± 4.05	−138.22 ± 3.05	−2.08 ± 0.10
2.0%CMC	618.13 ± 12.4^b^	18.87 ± 7.1	−145.09 ± 6.01	−2.05 ± 0.81
1%HPMC	418.40 ± 14.1^e^	2.47 ± 1.07	−182 ± 4.39	−2.61 ± 0.26
1.5%HPMC	516.36 ± 8.52^c^	1.65 ± 0.03	−26.22 ± 0.43	−2.41 ± 0.99
2.0%HPMC	460.75 ± 8.98^d^	1.96 ± 0.10	−184.29 ± 3.40	−2.55 ± 0.85

Different letters in each column indicate statistical differences (*P *≤* *0.05). Rice flour (RF), wheat flour (WF), and flour blend (FF) were controls and were made of 100%RF, 100%WF, and formulated FF, respectively.

The film‐like veil witnessed in the SEM scans of the treatment samples in Figure 1 and believed to be formed by hydrocolloids, can partially explain the increase in fracture force and hardness. As these films dehydrate during the baking process, they create a type of tough skin at the surface of the cracker which increases its resistance to breakage and thus increases the fracture force and hardness values. The hydrocolloids also helped retain gas in samples during baking so the crispness of gluten‐free crackers with hydrocolloids did not rely only on starch gelatinization as in the case of RF.

The addition of the hydrocolloids results in an increase in the rigidity as a consequence of the decrease in the swelling of starch granules and amylose lixiviation (Biliaderis et al. 1997). In addition as Rosell et al. (2001) found the addition of xanthan increased the hardness of the bread crumbs which could be a consequence of the thickening effect of hydrocolloids on the crumb walls surrounding the air spaces.

The microstructures of gluten‐free cracker dough were obtained by performing SEM analysis with 700× magnifition and 1500× magnifition (Fig. 1). Compared to the 100%RF control, samples made with hydrocolloids displayed a more irregular starch matrix structure, with the starch granules appearing somewhat deformed. However, not all starch granules lost their identity and they did not disintegrate completely.

Dough made from the two negative controls RF & FF tended to be porous and to have gaps of varying sizes and frequency within their matrix, whereas the WF positive control had a continuous matrix with no visible gaps. The dough microstructure of samples containing hydrocolloids had a more continuous matrix than the negative controls RF & FF. Hydrocolloids‐added dough seemed to hold the constituent starch granules and matrix covering them within a veil‐like film.

These findings agree with the result that Bárcenas and Rosell (2005) reported that the gas cell walls of the crumb‐containing HPMC showed a smooth structure with a fewer number of cavities than wheat bread without HPMC. They had a continuous structure with the appearance of a veil where the bread components could be observed. In addition, the use of hydrocolloids, such as XN in gluten‐free bread produces a web‐like structure similar to that of standard wheat bread (Ahlborn et al. 2005). However, the hydrocolloid‐containing samples were able to produce fairly stable gas cells and fairly continuous matrixes on their own without the addition of proteins (Fig. 1). These results appear to contradict others (Ahlborn et al. 2005; Rosell and Marco 2008) who had suggested that hydrocolloids alone do not seem to do enough to stabilize gas cells.

Table 3 shows the effect of different hydrocolloids and usage levels on the color components of rice crackers. The lightness values (L*) of all treatment samples were not significantly different when compared with formulated flour without hydrocolloids added (FF). The L* values of gluten‐free rice crackers containing 0.5%XN, 1.5%HPMC, and 1.5%CMC were the closest to WF; the rest of the treated samples had a lighter color and tended to be closer to FF.

**Table 3:** Effect of hydrocolloids on color values of gluten‐free rice crackers

Sample	Color		
	L*	Hue angle	Chroma
WF	45.8 ± 2.7^e^	78.6 ± 2.3^f^	34.8 ± 2.1^a^
RF	51.6 ± 1.5^a,b,c^	85.1 ± 1.2^a^	32.6 ± 1.2^d^
FF	50.0 ± 2.0^d^	85.3 ± 1.1^a^	31.7 ± 1.1^e^
0.25%XN	50.7 ± 1.8^c,d^	81.6 ± 2.0^e^	34.1 ± 1.5^a,b^
0.5%XN	49.7 ± 1.5^d^	83.9 ± 1.7^b,c^	34.2 ± 1.6^a,b^
0.75%XN	51.6 ± 1.3^a,b,c^	83.6 ± 2.0^c,d^	34.3 ± 1.4^a,b^
1.0%CMC	51.5 ± 2.3^a,b,c^	85.0 ± 2.3^a^	33.1 ± 1.8^c,d^
1.5%CMC	51.1 ± 2.0^b,c^	84.7 ± 2.1^a,b^	31.8 ± 1.2^e^
2.0%CMC	51.8 ± 2.5^a,b^	84.6 ± 2.3^a,b^	32.7 ± 1.4^d^
1%HPMC	52.5 ± 2.3^a^	85.4 ± 1.5^a^	34.0 ± 1.5^a,b^
1.5%HPMC	50.5 ± 2.3^c,d^	84.0 ± 1.9^b,c^	33.6 ± 1.6^b,c^
2.0%HPMC	51.3 ± 2.2^b,c^	82.9 ± 1.8^d^	34.3 ± 2.1^a,b^

Different letters in the same column indicate statistical differences found on each parameter of color (*P ≤ *0.05).

**Figure 2:**
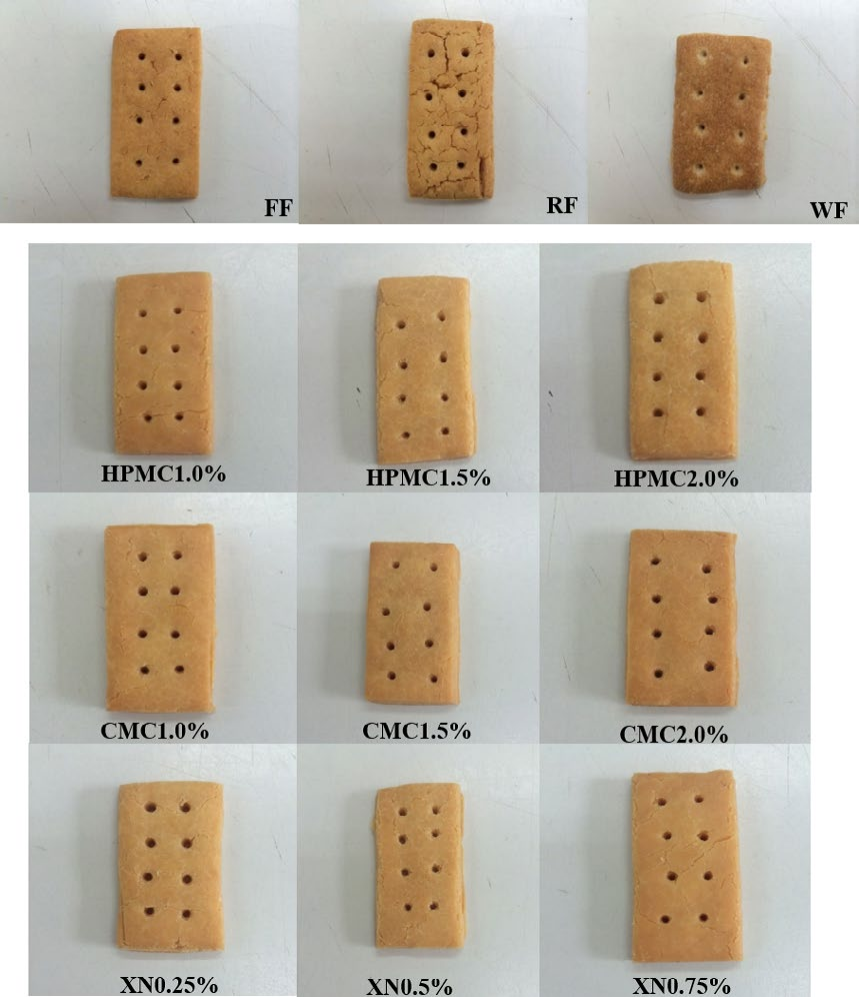
Effect of hydrocolloids on surface appearance of gluten‐free rice crackers. Rice flour (RF), wheat flour (WF), and flour blend (FF) were controls and were made of 100%RF, 100%WF, and formulated FF, respectively.

The surface color of 100% wheat crackers (WF) was more reddish‐brown than yellow‐brown, whereas 100% rice crackers (RF) had a more pale yellow character. Inclusion of XN and HPMC produced treated samples with similar yellowness (b*) and color intensity (Chroma) to the WF control. Despite the color values obtained by instrumental methods, all crackers evaluated showed slight differences in color and surface appearance from each other as illustrated in Figure 2. The darker color of the WF samples is due to a higher level of protein in the wheat samples which leads to higher amounts of free‐amino acids available to participate in maillard browning. Thus, samples made with wheat have a higher concentration of maillard browning reaction products which lead to a more reddish brownish color for these samples when compared to the treatment and negative control samples which have a lower level of proteins.

The addition of hydrocolloid to samples produced lighter colored crackers compared to the FF control. This can be explained by the increase in moisture content; as the moisture content increased, the Maillard browning reaction rate slowed and the reaction products responsible for brown color became further diluted thus producing a lighter finished cracker color (Mezaize et al. 2010).

### Effect of protein isolates on physicochemical properties of gluten‐free dough and crackers

The moisture content, water activity (*a*_w_), and % puffiness of samples with added protein isolates are shown in Table 4. The addition of different protein isolates increased the moisture content of gluten‐free crackers. Significant differences were observed in the moisture content of the treated samples (*P *≤* *0.05). The treated crackers samples containing 5% and 10% soy protein isolate had the highest moisture content of all samples measured. The moisture content of the treated crackers samples containing 5% and 10% whey protein isolates were closest to those of wheat crackers.

**Table 4:** Effect of protein isolates from different sources physicochemical properties of gluten‐free rice crackers

Samples	Moisture content (%)	Water activity (*a*_w_)	Puffiness (%)
WF	4.43 ± 0.040^c^	0.347 ± 0.005^a^	63.63 ± 2.80^a^
RF	2.80 ± 0.031^e^	0.134 ± 0.001^d^	28.00 ± 2.17^c^
FF	3.35 ± 0.087^d^	0.303 ± 0.007^b^	36.18 ± 1.29^b,c^
FF + 2.5%SP	5.11 ± 0.23^b^	0.221 ± 0.001^c^	41.58 ± 1.12^b^
FF + 5.0%SP	5.76 ± 0.37^a^	0.240 ± 0.008^c^	43.36 ± 2.45^b^
FF + 10.0%SP	5.84 ± 0.13^a^	0.318 ± 0.001^b^	44.42 ± 1.67^b^
FF + 2.5%PP	5.20 ± 0.16^b^	0.266 ± 0.001^c^	40.63 ± 4.22^b^
FF + 5.0%PP	5.29 ± 0.05^b^	0.248 ± 0.001^c^	41.77 ± 2.21^b^
FF + 10.0%PP	5.23 ± 0.07^b^	0.285 ± 0.001^b,c^	40.89 ± 1.34^b^
FF + 2.5%WP	5.29 ± 0.18^b^	0.319 ± 0.008^b^	43.78 ± 0.98^b^
FF + 5.0%WP	4.82 ± 0.122^b,c^	0.315 ± 0.002^b^	41.87 ± 1.67^b^
FF + 10.0%WP	4.62 ± 0.144^b,c^	0.251 ± 0.002^c^	42.08 ± 2.14^b^

Different letters in the same column indicate statistical differences (*P *≤* *0.05). Rice flour (RF), wheat flour (WF), and flour blend (FF) were controls and were made of 100%RF, 100%WF, and formulated FF, respectively.

The puffiness of all protein‐isolate‐treated samples appeared to be higher than controls made from RF were statistically significantly different (*P *≤* *0.05). The addition of protein isolates increased the puffiness of treated samples, but these samples were still less puffy than wheat crackers. The puffiness of wheat crackers was directly related to the protein matrix created in wheat crackers. The gluten matrix contains many inner layers which slows down the rate of gas diffusion and allows its retention before and after baking as well as determines the puffiness and crumb structure of the wheat crackers (Faubion and Hoseney 1990). In contrast, a continuous protein network with starch granule embedded was not found in the negative control (RF). Rice cracker texture was mostly created by starch gelatinization (Sozer 2009) and rice dough cannot retain the gas produced during fermentation which led to a crumbly rice cracker (He and Hoseney 1991). The addition of protein isolates to replace the gluten in the protein matrix produced protein matrixes with qualities between those of the wheat cracker and the negative control.

The texture characteristics of gluten‐free rice crackers are shown in Table 5. The addition of protein isolates decreased the fracture forces required to fracture the crackers when compared to the FF cracker so samples had a closer texture to wheat crackers. The hardness of samples containing 2.5 or 5.0% soy protein isolates and 5.0% pea protein isolates were close to those of wheat crackers (WF), and their texture characteristics were also similar to wheat crackers. In contrast, the hardness of the control rice cracker (RF) was the lowest, and samples were the most crumbly and dense, illustrated as lack of layers. This was due to the differences between rice protein and wheat gluten. Unlike wheat gluten, rice‐flour dough is not cohesive and lacks good viscoelastic properties and is not strong enough to entrap gas produced in the food system (Sozer 2009). Rice cracker structure is mostly created from starch gelatinization that cannot act as a strong backbone to support crackers' structure.

**Table 5:** Effect of protein isolates from different sources on texture characteristics of gluten‐free rice crackers

Samples	Attributes			
	Hardness (g)	Cohesiveness	Chewiness (gmm)	Springiness (mm)
WF	309.6 ± 7.9^f,g^	0.64 ± 0.02	1.04 ± 0.01	0.06 ± 0.04
RF	165.8 ± 5.7^k^	0.12 ± 0.05	11.6 ± 1.86	0.51 ± 0.03
FF	459.0 ± 4.97^c^	0.02 ± 0.01	0.14 ± 0.02	0.01 ± 0.00
FF+2.5%SP	297.8 ± 5.3^h^	0.16 ± 0.02	2.03 ± 0.03	0.32 ± 0.01
FF+5.0%SP	319.0 ± 5.0^f^	0.03 ± 0.04	6.53 ± 0.00	0.31 ± 0.02
FF+10.0%SP	226.5 ± 4.6^j^	0.03 ± 0.05	8.52 ± 0.00	0.19 ± 0.03
FF+2.5%PP	252.6 ± 6.0^i^	0.69 ± 0.01	2.03 ± 0.04	0.82 ± 0.004
FF+5.0%PP	320.2 ± 7.4^f^	0.12 ± 0.01	2.82 ± 0.01	0.71 ± 0.004
FF+10.0%PP	555.0 ± 28.6^b^	0.07 ± 0.02	1.88 ± 0.01	0.57 ± 0.003
FF+2.5%WP	402.2 ± 12.3^e^	0.17 ± 0.02	0.01 ± 0.001	0.02 ± 0.001
FF+5.0%WP	433.6 ± 6.32^d^	0.11 ± 0.03	0.05 ± 0.001	0.16 ± 0.001
FF+10.0%WP	794.6 ± 56.15^a^	0.15 ± 0.08	0.01 ± 0.002	0.23 ± 0.001

Different letters in the same column indicate statistical differences (*P *≤* *0.05). Rice flour (RF), wheat flour (WF), and flour blend (FF) were controls and were made of 100%RF, 100%WF, and formulated FF, respectively.

The formulated flour‐blended control (FF) contained pregelatinized‐tapioca starch in the FF that facilitated dough formation from more starch gelatinization (Sozer 2009), consequently resulting in better texture than rice control (Nammakuna et al. 2009). However, the texture of nonprotein‐added control still had fewer layers and was less puffy, which made its texture tougher and less brittle than wheat crackers, despite its lower values of hardness. Besides wheat crackers, the protein‐added samples were perceived as relatively more brittle and crisper than other controls because there were more layers and puffiness. These results agreed with (Sozer 2009) that addition of proteins helps starch granules to adhere to one another, and water is more distributed through the system because of the polymeric structure of proteins (Sivaramakrishnan et al. 2004).

The viscoelastic behavior of dough was illustrated in terms of storage modulus (G′), loss modulus (G″), and tanδ value. They were used as the indicators of dough rheology and characteristics. The loss tangent or tanδ is the tangent of the phase angle which is the ratio of viscous modulus (G″) to elastic modulus (G′) that shows the presence of fluid's elasticity. The loss tangent values less than unity indicate an elastic‐dominant behavior, whereas values greater than unity indicate viscous‐dominant behavior. Simply put, these values could be used to describe the balance between elastic properties such as film formation and gas retention in dough, and viscous properties such as protein absorption to the liquid lamella and flexibility for gas expansion in dough (Lazaridou et al. 2007).

**Figure 3:**
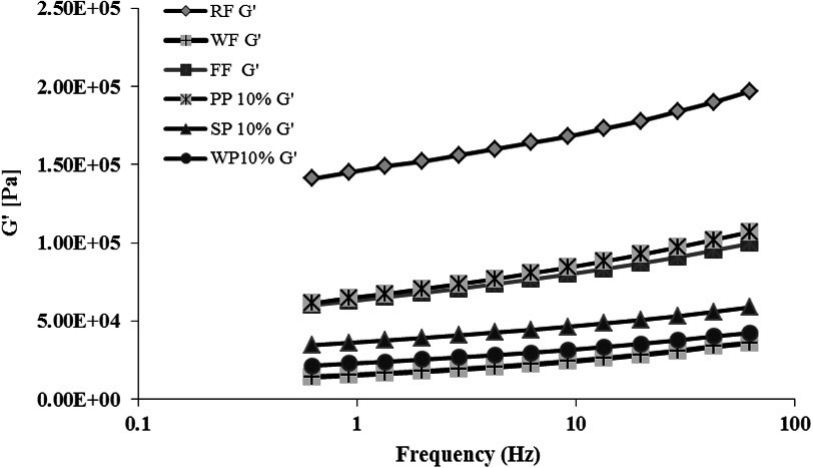
Effect of protein isolates from different source on rheological properties of gluten‐free rice cracker based on the frequency sweep test. Rice flour (RF), wheat flour (WF) and flour blend (FF) were controls and were made of 100%RF, 100%WF and formulated FF, respectively.

The viscoelastic behavior of dough samples with addition of protein isolates are illustrated in Figure 3 and Table 6. The control rice dough had the highest storage modulus value which made its structure more rigid and less elastic. This also made its structure harder to poor stretch during kneading and sheeting process. On the other hand, wheat dough with gluten network formation had the lowest storage modulus value which caused a unique viscoelastic property in dough, being more elastic, easier to handle in kneading, and in sheet‐ making (Rosell and Marco 2008).

**Table 6:** The effect of hydrocolloids and protein isolates from different source on storage modulus (G′), loss modulus (G″), and tanδ on gluten‐free rice cracker dough

Samples	Viscoelastic parameter (overall mean)		
	G′ (Pa)	G″ (Pa)	tanδ
WF	23538.46	10500.77	0.446
RF	95500.00	22884.62	0.239
FF	114700.00	28384.61	0.244
FF + 10%SP	45400.00	10766.15	0.237
FF + 10%SP + 0.5%XN	60276.92	16646.15	0.276
FF + 10%SP + 1.5%HPMC	110915.21	32318.87	0.264
FF + 10%PP	82030.77	19615.38	0.239
FF + 10%PP + 0.5%XN	52515.38	13040.77	0.248
FF + 10%PP + 1.5%HPMC	95812.93	23211.62	0.242
FF + 10% WP	30807.69	10586.92	0.344
FF + 10%WP + 0.5%XN	94684.61	28284.61	0.298
FF + 10%WP + 1.5%HPMC	99628.12	27587.95	0.277

Rice flour (RF), wheat flour (WF), and flour blend (FF) were controls and were made of 100%RF, 100%WF, and formulated FF, respectively.

In doughs made with protein isolates, the storage modulus values were lower than the control rice dough; in particular dough with a 10% whey protein added had a storage modulus closest to that of the wheat cracker dough (Fig. 3), showing an improvement in dough elasticity. This was probably due to the polymeric structure of protein that facilitated better water‐holding capacity and water distribution in dough when compared with rice control. Sozer (2009) also noted that a decrease in storage modulus value was observed in pasta dough with more gelatinized RF because of an increase in water absorption and starch granule swelling. The addition of protein isolates to replace the gluten in the protein matrix produced protein matrixes with qualities between those of the wheat cracker and the negative control.

Table 6 illustrates the loss tangent (tanδ) of dough samples with addition of protein isolates and hydrocolloids. All dough samples had tan delta values less than unity which means their behaviors were more elastic than viscous. Among all samples, wheat crackers had the highest loss tangent value (0.44) which indicated more elasticity than others. As compared, among the other protein‐isolate dough samples, the loss tangent values of dough with whey protein isolates were closer to wheat dough. Those values were also higher than dough of RF or FF, indicating samples were more elastic and stretchy. This behavior could be explained that the addition of protein isolates increased the amount of polymers in the dough system, resulting in an improvement in elastic properties of dough samples (Sozer 2009).

### Effect of protein isolate and hydrocolloids on physicochemical properties of gluten‐free dough and crackers

The effects of hydrocolloids and protein isolate combinations on the physical and chemical properties of gluten‐free crackers are shown in Table 7. Significant differences in moisture content and water activity (*a*_w_) in all treatments were observed (*P *≤* *0.05). The addition of hydrocolloids and protein isolates produced moisture contents and water activity (*a*_w_) values higher than that of the formulated flour‐ blended control (FF), with the exception of the 1.5%HPMC and 10% soy protein sample which was not significantly different from the formulated FF control.

**Table 7:** Physicochemical properties of gluten‐free rice crackers with protein isolates from different sources and hydrocolloids addition

Samples	Moisture content (%)	Water activity (*a*_w_)	Puffiness (%)
WF	4.43 ± 0.040^c^	0.347 ± 0.005^a^	63.63 ± 4.80^a^
RF	2.80 ± 0.031^e^	0.134 ± 0.001^g^	28.00 ± 2.17^c^
FF	3.35 ± 0.087^d^	0.303 ± 0.007^d^	38.18 ± 3.29^b^
FF + 10%SP + 0.5%XN	4.96 ± 0.049^b^	0.324 ± 0.002^c^	42.54 ± 3.04^b^
FF + 10%SP + 1.5%HPMC	3.76 ± 0.123^d^	0.287 ± 0.002^f^	41.35 ± 2.47^b^
FF + 10%PP + 0.5%XN	5.28 ± 0.040^a^	0.337 ± 0.002^b^	41.81 ± 1.14^b^
FF + 10%PP + 1.5%HPMC	4.42 ± 0.100^c^	0.298 ± 0.006^e^	41.31 ± 1.79^b^
FF + 10%WP + 0.5%XN	4.88 ± 0.016^b^	0.331 ± 0.003^b^	40.45 ± 2.01^b^
FF + 10%WP + 1.5%HPMC	4.67 ± 0.040^b,c^	0.318 ± 0.005^c^	41.76 ± 1.73^b^

Different letters in the same column indicate statistical differences (*P *≤* *0.05). Rice flour (RF), wheat flour (WF), and flour blend (FF) were controls and were made of 100%RF, 100%WF, and formulated FF, respectively.

The treated samples with 10% pea protein isolate and 0.5%XN had the highest moisture content and water activity (*a*_w_). The addition of 1.5%HPMC in the samples containing 10% pea protein isolate and 10% whey protein isolate caused the moisture content to become closer to that of wheat crackers and higher than the FF control.

This resulted in water retention ability of this dough due to its hydrophilic nature (Christianson et al. 1981; Twillman and White 1988; Bell 1990; Dziezak 1991; Armero and Collar 1998; Gurkin 2002; Guarda et al. 2004). The hydrocolloids in the dough held on to a fraction of the water. These findings are consistent with prior research on the effects of hydrocolloids on the properties of baked bread loaves, for example, as reported by Guarda et al. (2004), the HPMC network formed during baking could act as a barrier to gas diffusion, decreasing the water vapor losses, and increasing the final moisture content of the loaves. However, the addition of 1.5%HPMC into dough containing 10% soy protein isolate and 10% pea protein isolate produced a decrease in the moisture content of the samples compared to dough containing only 10% pea protein and 10% soy protein. This effect can be attributed to the unique interaction between HPMC and soy protein according to Rosell and Marco (2008), the reduction in the moisture content induced by the HPMC was partially masked, when part of the RF was replaced by soybean protein, where rice starch molecules were replaced by protein molecules. No significant difference in puffiness was found among treatments (*P *>* *0.05). The hydrocolloids‐added samples showed higher puffiness than control formulated flour (FF) and 100%RF.

**Figure 4:**
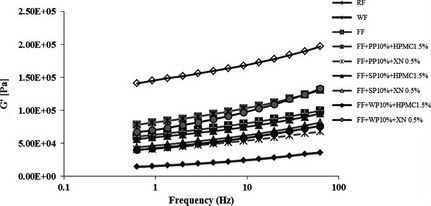
Effect of protein isolates from different sources and hydrocolloids addition on rheological properties of gluten‐free rice crackers based on the frequency sweep test. Rice flour (RF), wheat flour (WF) and flour blend (FF) were controls, and were made of 100%RF, 100%WF, and formulated FF, respectively.

The texture characteristics of the combinations of protein isolates and hydrocolloids are shown in Table 8. The hardness of all treatment samples was higher compared to RF and FF control (*P *≤* *0.05), thus the samples were less crumbly and crispier. These results agreed with previous studies which determined that hydrocolloids such as HPMC increased water binding in the rice cassava dough, and the amphiphilic nature of the hydrocolloids acted as a surfactant stabilizing the gas–liquid interface around the gas bubble and resulting in increased loaf volume, improved crumb structure, and reduced crumb firmness (Crockett et al. 2011). The viscoelastic behavior of protein and hydrocolloids‐added dough samples was investigated by the oscillation frequency sweep test using a frequency sweep from 0.1 to 10 Hz. The results are shown in Figure 4.

**Table 8:** The texture characteristic of gluten‐free rice crackers with protein isolates from different sources and hydrocolloids addition

Samples	Attributes			
	Hardness (g)	Cohesive	Chewiness (gmm)	Springiness (mm)
WF	336 ± 6.24^d^	0.066 ± 0.014	4.14 ± 0.012	1.09 ± 0.008
RF	68.75 ± 2.68^g^	0.122 ± 0.05	11.59 ± 15.86	0.81 ± 0.010
FF	401.75 ± 7.60^b^	0.069 ± 0.07	21.89 ± 0.018	2.51 ± 0.012
FF + 10%SP + 0.5%XN	244.50 ± 5.42^f^	0.53 ± 0.013	5.03 ± 0.002	4.18 ± 0.034
FF + 10%SP + 1.5%HPMC	278.0 ± 5.55^e^	0.66 ± 0.023	6.13 ± 0.002	6.09 ± 0.018
FF + 10%PP + 0.5%XN	332.0 ± 2.56^d^	0.78 ± 0.044	4.93 ± 0.002	3.22 ± 0.001
FF + 10%PP + 1.5%HPMC	274.5 ± 5.89^e^	0.18 ± 0.050	3.02 ± 0.002	2.39 ± 0.001
FF + 10%WP + 0.5%XN	358.4 ± 8.20^c^	0.18 ± 0.034	6.22 ± 0.002	3.32 ± 0.002
FF + 10%WP + 1.5%HPMC	454.8 ± 5.02^a^	0.12 ± 0.008	7.23 ± 0.002	3.11 ± 0.002

Different letters in the same column indicate statistical differences (*P *≤* *0.05). Rice flour (RF), wheat flour (WF), and flour blend (FF) were controls and were made of 100%RF, 100%WF, and formulated FF, respectively.

All samples showed an increase of the storage modulus (G′) with increasing frequency. The storage modulus of rice cracker dough was the highest followed by dough samples with hydrocolloids. In dough containing both soy protein isolate and HPMC, the protein altered the HPMC functionality due to competition for water. This weakened the HPMC interactions with the starch matrix and reduced form stability (Crockett et al. 2011). Addition of 0.5%XN resulted in dough with viscoelastic properties close to 100% wheat dough, especially cracker dough containing 10% pea protein and 0.5%XN, thus dough had G′ values closer to 100% wheat dough than all other treatments.

The dough‐containing combinations of hydrocolloids and protein isolates had tanδ values higher than 100% rice cracker dough and formulated flour dough controls (Table 6). The dough with 10% whey protein isolate had the highest tanδ values, followed by a combination of 0.5%XN and 10% whey protein isolated sample, respectively. The G′ values of all treatment samples were slightly increased, and the dough became more rubbery when hydrocolloids were added which facilitated better dough kneading and sheeting when compared to the RF dough. According to (Sánchez et al. 2004), this may have been caused by a specific interaction between proteins and hydrocolloids leading to the formation of a more viscoelastic dough. The addition of hydrocolloids into protein isolates‐treated doughs caused the G′ values to increase for all treatments, especially when HPMC was added to soy protein isolates and pea protein isolates dough, which had G′ values close to formulated FF dough. The addition of hydrocolloids to leguminous protein isolate‐treated dough induced an increase in the loss tangent (tanδ). Crockett et al. (2011) also found that the addition of hydrocolloids and soy protein isolate increased G′ in gluten‐free bread from rice and cassava which may have been caused by the action of the two main globulins in soy protein isolate, including β‐conglycinin and glycinin (Lampart‐Szczapa 2001; Crockett et al. 2011). Glycinin forms a thermoplastic gel at 80°C, but above 100°C, the further unfolding of protein exposes more hydrophobic regions, further stabilizing the gel and preventing denaturing of the protein (Lampart‐Szczapa 2001).

## Conclusion

In crackers, wheat gluten plays a very unique role compared to its functionality in other baked products as it acts to make the cracker weaker and stronger at the same time. While the strong protein structure created by gluten–protein interaction creates rigid, crispy, and continuous layers in the cracker, at the same time it aides in the formation of a relatively small number of relatively large gas bubbles, which separates these layers thus reducing the density of the overall cracker and increasing its overall surface area, thus increasing the moisture loss rate, and therefore resulting in a low moisture, crispy finished texture.

Addition of protein isolates helps improve the performance of gluten‐free rice crackers, with the amount of improvement being dependent on the nature and the functional properties of the added protein. The gluten in wheat‐based crackers is able to form elastic structures that entrap air and expand to relatively large sizes without bursting during the baking process. As a result, crackers made with WF have a comparatively small number of large gas cells, whereas dough made with rice FF has a high number of relatively small gas cells as the dough cannot produce the same type of elastic structures found in wheat‐based crackers, due to significantly lower levels of protein in RF and thus cannot produce large gas cells. The microstructure of dough‐containing hydrocolloids had a more continuous matrix with fewer and larger gas cells when compared to FF and 100% rice crackers, the dough seemed to hold the constituent, starch granules, and matrix covering resembling a veil‐like film.

Cracker doughs made with hydrocolloids and protein isolates have structures containing gas cell numbers and sizes in between those seen in 100% wheat crackers and those seen in the rice‐blended crackers. This more compact structure combined with the higher water‐holding ability of hydrocolloids lead to higher moisture contents in the finished cracker. This higher finished moisture content and the lower protein content, compared to 100%WF crackers, resulted in lighter colored crackers. The added hydrocolloids and protein isolates interacted with the native proteins of the rice formula to create more elastic structures that could partially mimic those created by the gluten‐forming protein in wheat‐based crackers. The combination of hydrocolloids and protein isolates create cracker dough which have higher G′ values and are more rubbery than dough made from the FF blend, this facilitates dough kneading and sheeting as it forms a more viscoelastic gel. However, soy protein isolates and pea protein isolates lead to undesirable flavors in the finished cracker.

Thus, this research shows that combinations of hydrocolloids and protein isolates can be used to partially or fully replace wheat gluten in crackers with acceptable organoleptic results, however, no combination produced gluten‐free crackers with organoleptic properties identical to those of wheat gluten‐containing crackers. The cracker containing 10.0% whey protein isolate had the best texture characteristics and rheological properties closest to the 100% wheat control.

## Conflict of Interest

None declared.
